# Double masking protection vs. comfort—A quantitative assessment

**DOI:** 10.1063/5.0058571

**Published:** 2021-07-21

**Authors:** Venugopal Arumuru, Sidhartha Sankar Samantaray, Jangyadatta Pasa

**Affiliations:** Applied Fluids Group, School of Mechanical Sciences, Indian Institute of Technology Bhubaneswar, Bhubaneswar 752050, India

## Abstract

COVID-19 has forced humankind to adopt face masks as an integral part of everyday life. This preventive measure is an effective source control technique to curb the spread of COVID-19 and other similar diseases. The virus responsible for causing COVID-19 has undergone several mutations in the recent past, including B.1.1.7, B.1.351, P.1, and N501Y, B.1.617, with a higher infectious rate. These viruses' variants are mainly responsible for the recent spike in COVID-19 cases and associated steep rise in mortality rate worldwide. Under these circumstances, the Center for Disease Control (CDC) and health experts recommend double masking, which mainly includes a surgical mask and a cotton mask for the general public. This combination provides an additional layer of protection and masks fitment to minimize the leakage of droplets expelled during coughing, sneezing, talking, and breathing. This leakage may cause airborne transmission of the virus. In the present study, we report a systematic quantitative unsteady pressure measurement supplement with flow visualization to quantify the effectiveness of a single and double mask. We have also evaluated double masking consisting of a surgical mask and an N-95 mask used by medical professionals. A simple knot improves the surgical mask fitment significantly, and hence, the leakage of droplets is minimized. The leakage of the droplets was reduced to a large extent by using a double mask combination of a two-layer cotton mask over the surgical mask with a knot. The double mask combination of surgical + N-95 and two-layer cotton + N-95 masks showed the most promising results, and no leakage of the droplets is observed in the forward direction. A double mask combination of surgical and N-95 mask offers 8.6% and 5.6% lower mean and peak pressures compared to surgical, and cotton mask. The best results are observed with cotton and N-95 masks with 54.6% and 23% lower mean and peak pressures than surgical and cotton masks; hence, this combination will offer more comfort to the wearer.

## INTRODUCTION

COVID-19 pandemic is one of the toughest social, economic, and technological challenges humankind has encountered in the last ten decades. After a prolonged lockdown period, many countries have started regular activities, which has imparted some boon to the economy. However, some new normals like a face mask and social distancing have become an integral part of day-to-day activities. The recent spike in COVID-19 cases and associated steep rise in mortality rate worldwide raises questions about the complete fundamental understanding of various possible modes of virus transmission. In the recent past, scientists have proposed various mitigation measures,^1–14^ explained virus survival,^15–21^ disinfecting strategies for surfaces, and protective measures efficacy.^22–29^ Social distancing guidelines have also been proposed considering extreme events like coughing and sneezing.^30–33^ For the general public, the policymakers have framed guidelines to curb the spread of COVID-19; this mainly includes a face mask,^34^ social distancing,^35^ and frequent handwash.^36^ The second and the third wave of COVID-19 in many countries is much more severe in terms of infection spreading rate and mortality. One possible reason for this is new variants of the virus (B.1.1.7, B.1.351, and P.1), which are more infectious.^24,37^

The face mask has become an integral part of everyday life in the present COVID-19 scenario. The effectiveness of various single masks under the influence of coughing,^26–29,38^ sneezing,^23,24^ and breathings^39–41^ are reported in the recent past. The leakage of smaller droplets responsible for airborne transmission of the virus is a concern from various single masks, primarily due to mask fitment.^24,41^ The leakage of droplets also depends on the type of mask and the velocity of the expelled air during various respiratory events. During coughing, the velocity of expelled air is 6–22 m/s,^42^ whereas, for breathing, it is restricted to 2.2–9.9 m/s.^43^ The droplets may reach up to 2.5 ft from a surgical mask during sneezing,^24^ whereas during breathing, the reach is 0.8 ft.^41^ Commonly used two-layer cotton masks are unable to impede the leakage of the droplets. The reach of the leaked droplet is reported as 1.5 ft during sneezing.^24^ This leakage is restricted to 0.4 ft during breathing.^41^ Hence, single masks are unable to limit the leakage of the droplets effectively.

Several mutants of the virus are noticed in the recent past, which are more infectious.^37^ Under these circumstances, more effective preventive measures are needed. A recent study conducted by Brooks *et al.*^44^ demonstrated that double masking cloth mask over a surgical mask reduces a wearer's exposure to the aerosolized particles by 90%. Brooks *et al.*^44^ used a coughing simulator and headform to simulate the penetration of aerosol particles from the various mask and evaluated the particle filtration efficiency. The Center for Disease Control (CDC) also recommends double masking to curb the airborne spread of COVID-19.^45^ Double masking provides an additional layer of protection and mask fitment to minimize the leakage of droplets expelled during coughing, sneezing, talking, and breathing. This leakage may cause airborne transmission of the virus.^38,46^

In the present work, we evaluate double-masking efficacy further with unsteady pressure measurement to quantify the pressure drop associated with the blockage to airflow created by double masking and flow visualization experiments to document the particles' reach and leakage visually. Such a study is not reported in the literature. The unsteady pressure measurement quantifies the resistance offered by double masking in terms of peak pressure and mean pressure, which is useful for assessing the physiologic and psychologic adverse health effects associated with long-term mask usage and wearing comfort. The flow visualization experiments qualitatively supplement the pressure measurements to demonstrate double-masking effectiveness with visual evidence, which is useful for spreading awareness in the general public. Under the present pandemic scenario, the general public is concerned about more effective preventive measures. Hence, such a study will be useful to establish more scientific evidence to promote double masking for the general public.

## EXPERIMENTAL SETUP

**FIG. 1.**
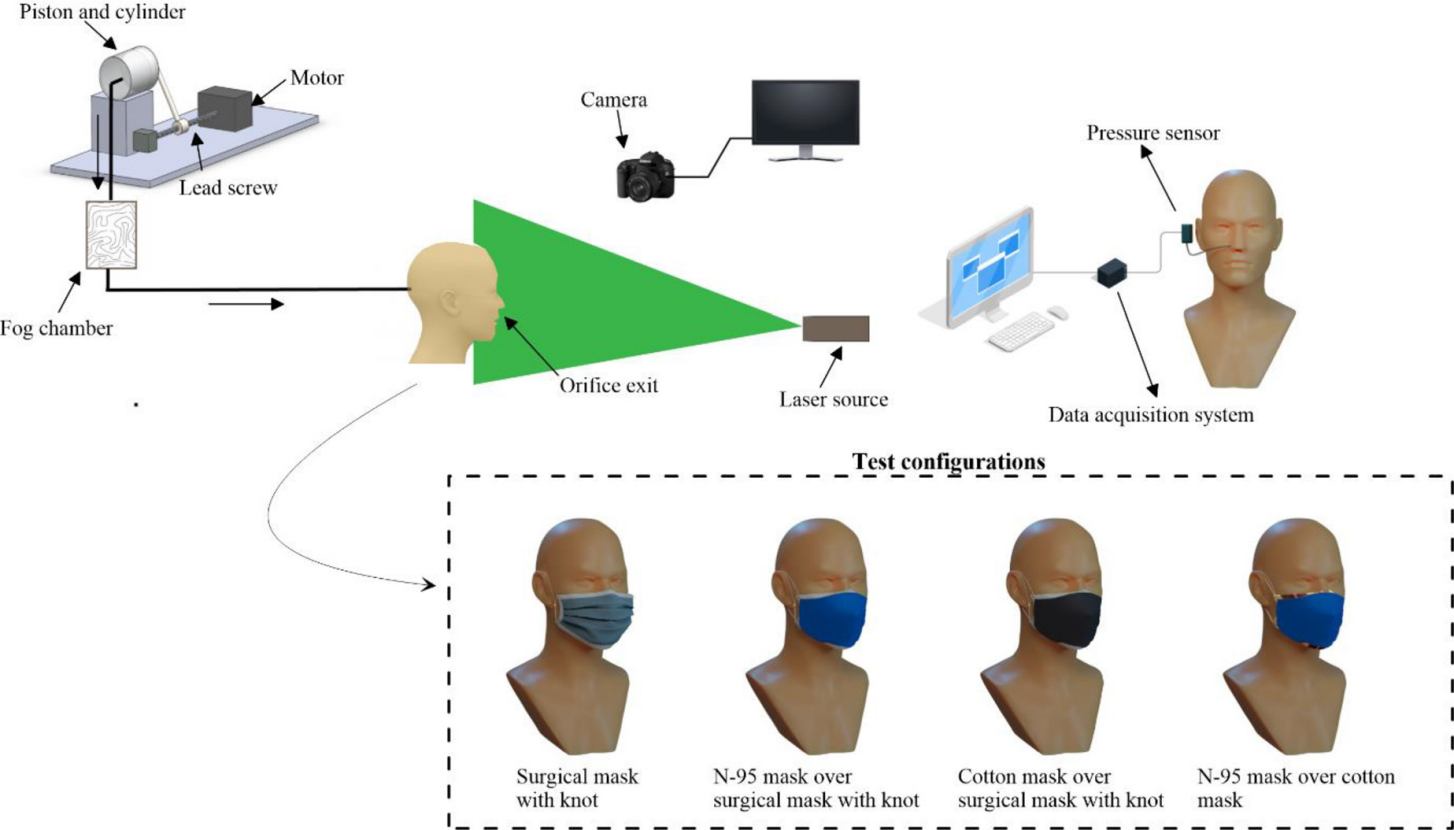
Experimental test facility (surgical and N-95 mask used in the present study are standard medical procedure mask, whereas five-layer mask is a standard antipollution mask and cotton mask is a nonstandard 0.45 + 0.05 mm thick mask with 55 threads/in. Knot in the surgical mask is created as per CDC guidelines^45^).

In the present study, we employ a breathing simulator fitted to a headform. The breathing simulator consists of a stepper motor and a piston-cylinder arrangement. The stepper motor can be appropriately programmed to achieve different exhale to inhale ratios, E:I. Our previous study validated and employed the breathing simulator to quantify breath reach for different exhale to inhale ratios and the efficacy of various protectives measures.^41^ In the present study, we employed the same system to quantify the unsteady static pressure generated due to various combinations of double masking and visualization of leaked droplets. The nose of the headform is fitted with a nozzle of diameter 10 mm, which is similar to a typical nostril opening area. A 1.5 mm static pressure tap is connected to the nozzle, as shown in Fig. 1.

An unsteady GREYSTONE make differential pressure sensor (LP3-A-04) with configurable differential pressure range ±250, ±500, and ±1000 Pa is employed to measure the unsteady static pressure generated due to the blockage imposed by various masks for different exhale to inhale ratios. The uncertainty in pressure measurement is estimated as ±1.5%. The response time of the sensor is 250 ms which is suitable for unsteady pressure measurements. The voltage output of the pressure sensor is logged to a personal computer using National Instruments (NI) data aquation system. In the present study, a breathing frequency of 15 BPM (Breath Per Minutes) is simulated with different exhale to inhale ratios (E:I = 1:1 and 1:2), which is a typical breathing frequency of a healthy adult.^47^ The breathing simulator displaces an average tidal volume of ∼500 ml corresponding to a healthy adult.^48^ Due to the curvature of the face of the headform, it was practically not possible to flush mount the pressure sensor. Hence, the pressure sensor is connected to the pressure tap with a nondeformable plastic tube of diameter 2 mm and length of 10 cm; the amplitude and phase distortion due to the connecting tube a negligible at these low frequencies.^49^ For flow visualization experiments, we employ a laser sheet with a digital camera and fog consisting of distilled water (70%) and glycerin (30%) as a tracer. The estimated diameter of the tracer droplets is <10 *μ*m, which represents aerosol droplets.^26,38,41^ The experiments are conducted in a quiescent environment.

The primary objective of any face mask is to prevent the leakage of droplets/particles during exhalation, coughing, sneezing, and talking and to filter the external droplets/particles during inhalation. However, breathability and wearing comfort are other aspects that need to be accounted for in an effective face mask design. The differential pressure across the face mask is a measure of breathability.^50^ The differential pressure is typically measured under steady flow conditions perpendicular to the plane of the face mask material. For homogeneous material, this differential pressure is directional independent; however, for inhomogeneous material and asymmetric mask design, the differential pressure depends on airflow direction.^50^

In the present study, we propose an alternate technique to evaluate the breathability by measuring the unsteady pressure drop across different masks fitted to a headform face. The different face masks are appropriately fitted to ensure a snugly fit to avoid any abrupt leakage and hence may be considered as a natural fit in the actual scenario. The added advantage of the present technique is the quantification of mean and peak pressure during exhalation and inhalation for different E:I ratios, which is unquantifiable using standard technique under steady flow conditions. Since a snugly fit face mask of various designs is not symmetric with flow direction due to the curvature of the face and flexibility of the face mask material, hence the proposed technique closely resembles the actual scenario. Therefore, the measured mean and peak pressure for various face mask combinations may be valuable to assess the breathability in the actual scenario. In the present COVID-19 pandemic situation, the most general public has adopted universal masking as a source control technique.

Moreover, double masking is gaining popularity in recent times due to its enhanced protection. However, the pressure drop associated with double masking for a prolonged period may cause physiologic and psychologic burden, and work efficiency may deteriorate in indoor environments due to discomfort.^51^ Face mask comfort is typically assessed with the pressure drop, and a lower pressure drop indicates breathing comfort.^52^ Higher pressure drop associated with face mask alleviates suffocation and hence not desirable.^53^ Hence, such a study may serve to establish the effectiveness of double masking from both filtration and breathability/comfort perspectives.

## RESULTS AND DISCUSSION

**FIG. 2.**
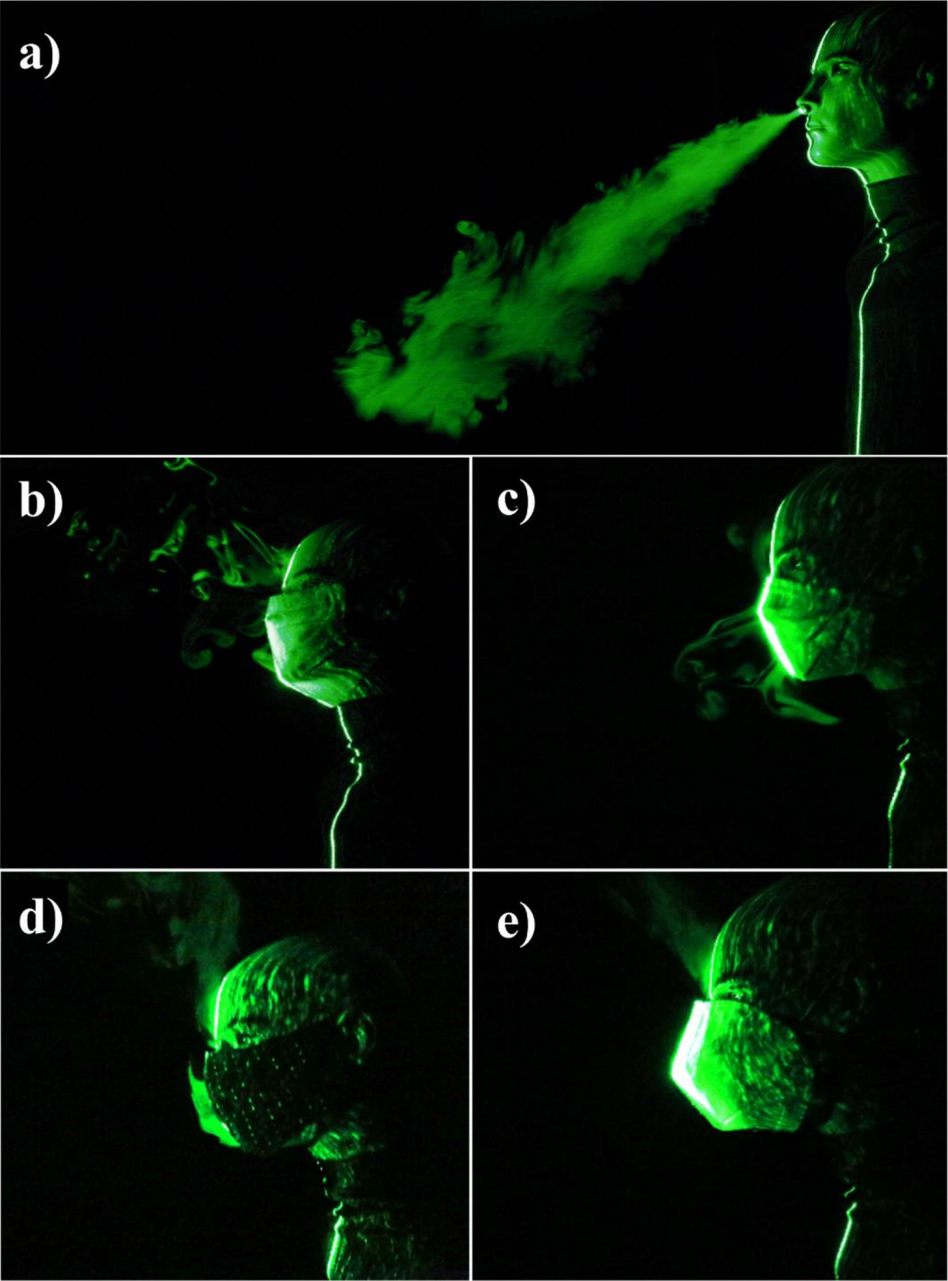
Droplet leakage from various masks (a) no mask (b) surgical mask (c) surgical mask with knot (d) double mask: surgical + cotton (e) double mask: surgical + N-95.

**FIG. 3.**
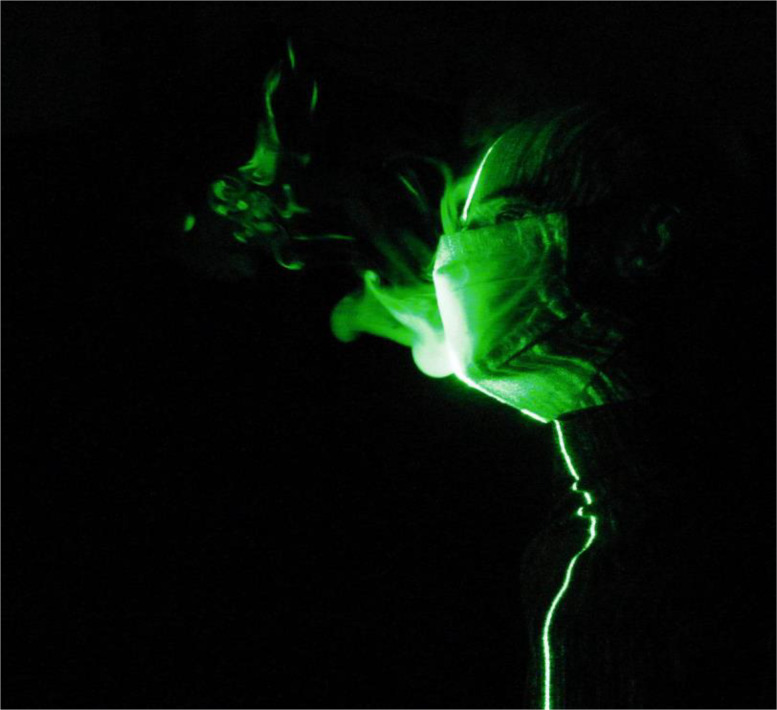
Snapshot of droplet leakage from surgical mask for E:I = 1:1. Multimedia view: https://doi.org/10.1063/5.0058571.1
10.1063/5.0058571.1

**FIG. 4.**
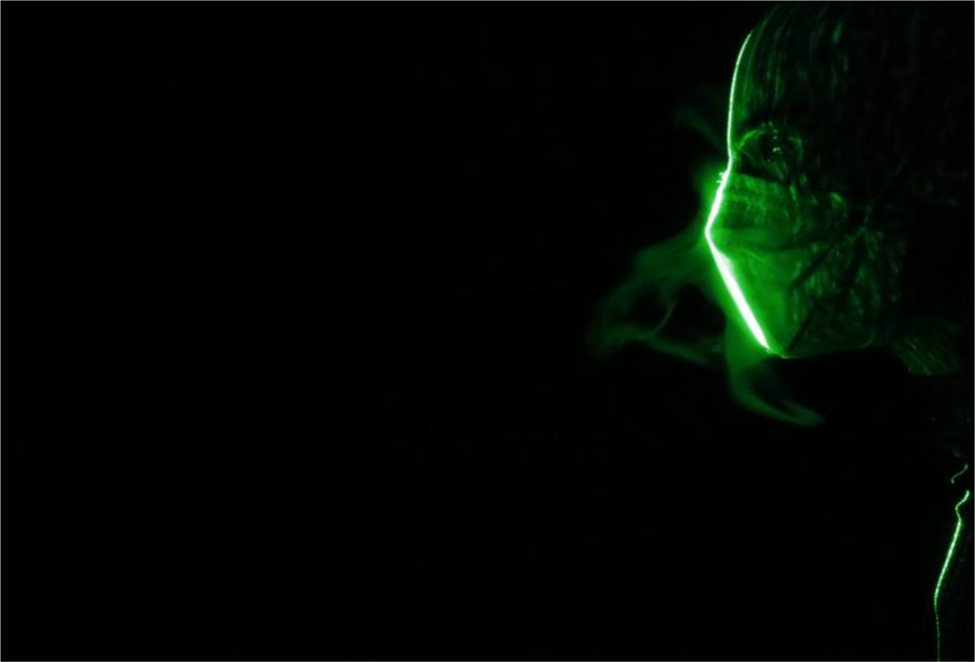
Snapshot of droplet leakage from a surgical mask with a knot for E:I = 1:1. Multimedia view: https://doi.org/10.1063/5.0058571.2
10.1063/5.0058571.2

**FIG. 5.**
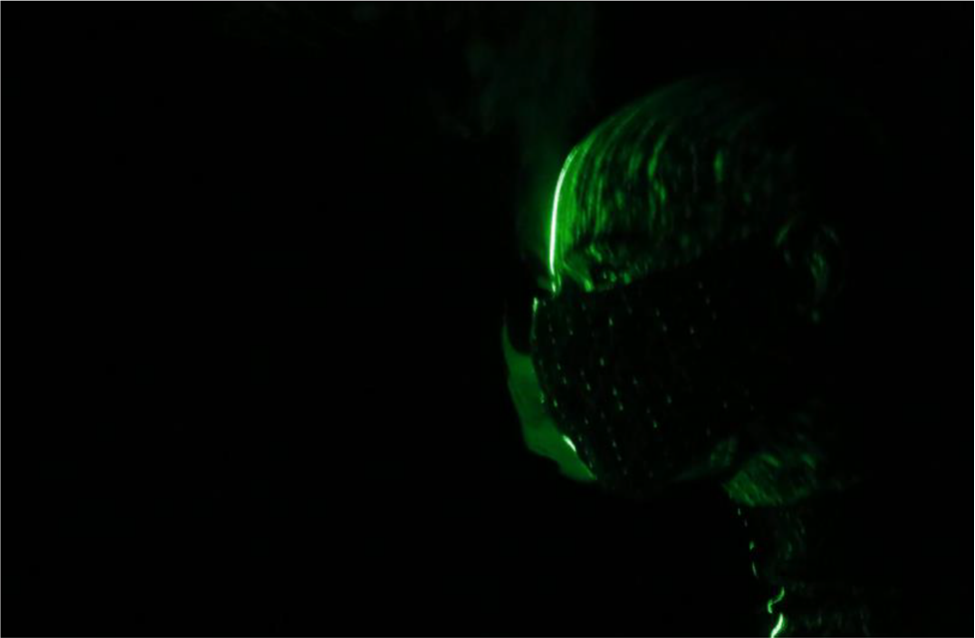
Snapshot of droplet leakage from surgical + cotton mask for E:I = 1:1. Multimedia view: https://doi.org/10.1063/5.0058571.3
10.1063/5.0058571.3

**FIG. 6.**
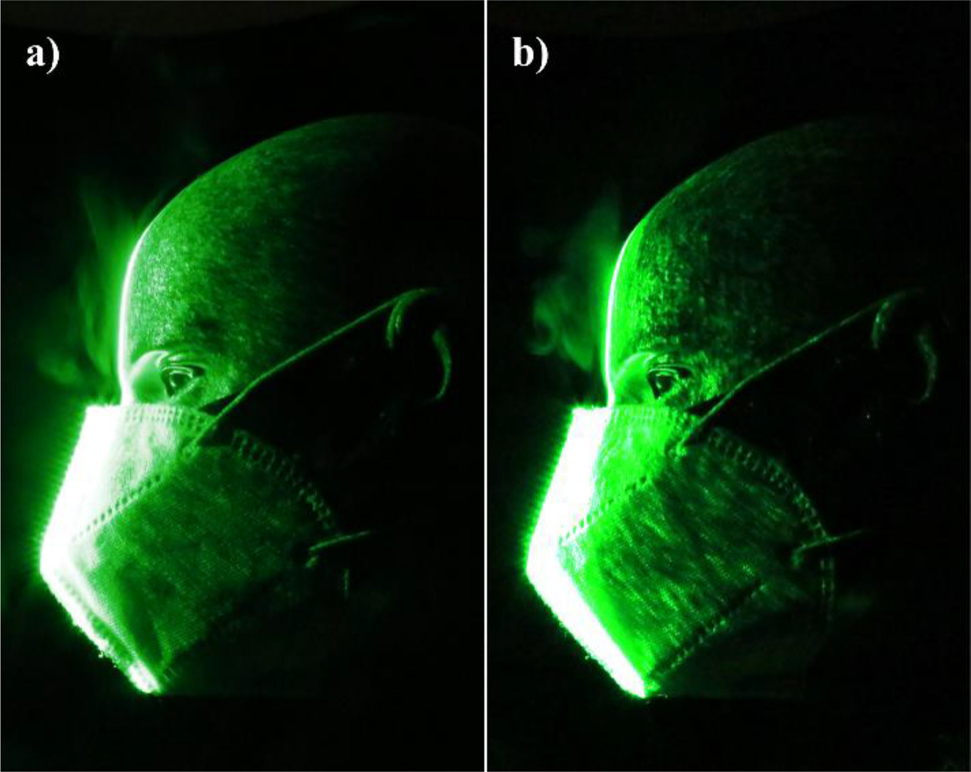
Droplet leakage from double mask: cotton + N-95.

The efficacy of double masking is studied qualitatively using flow visualization experiments. The leakage of the droplets from various masks and mask combinations (double masking) is shown in Fig. 2. The leakage of the droplets is significant from a surgical mask [Figs. 2(a) and 3]; this is mainly due to poor fitment. The standard surgical mask employed in the present study is equipped with a nose wire to improve the mask fitment near the nose; however, significant leakage of droplets is still observed. The surgical mask with a knot showed substantial improvement in preventing the leakage. A simple knot improves the surgical mask fitment significantly, and hence, the leakage of droplets is minimized [Figs. 2(b) and 4]. Our flow visualization study substantiates the findings reported by Brooks *et al.*^44^ The leakage of the droplets is reduced to a large extent by using a two-layer cotton mask over the surgical mask with a knot [Fig. 2(c) and 5]. The double mask combination of surgical and N-95 showed the most promising results, with no leakage of the droplets is observed in the forward direction; however, minimal leakage is observed in the upward direction near the nose [Fig. 2(d)]. Hence, attention needs to be paid to improve mask fitment on the nose to avoid this leakage. Individuals may have to adjust the position of the nose wire to improve the mask fitment further. No significant changes in the leakage are observed for different E:I ratios as shown in Fig. 6. The hydrodynamics of the droplets is influence by the addition of a second mask. In the case of a surgical mask, significant leakage is observed from the front and opening from the nose. The escaped droplets merge and move forward [Fig. 2(a)]. The leakage from the nose opening is reduced with the knot, and the droplets escape predominantly from the front. Their reach is also restricted compared to without knot case. The addition of a cotton mask over the surgical mask results in a reduction in the velocity of the escaped droplets from the front. The leaked droplets rise under the influence of buoyancy in the upward direction as a thermal plume [Fig. 2(c)]. However, the droplet's reach is restricted compared to the single mask case.

**FIG. 7.**
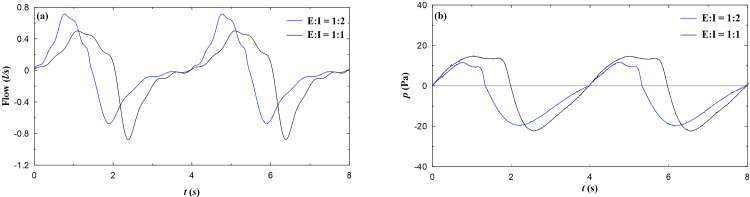
Breathing flow and static pressure profiles for various E:I ratio (a) volume flow variation with time (b) static pressure variation with time.

Unsteady pressure measurements are carried out for various single and double masks to quantify the pressure drop associated with the airflow blockage created by masks. The pressure drop is an indicator of breathability, a lower pressure drop indicates ease in breathing, and hence it is a desirable feature of any face mask.^52^ In the present study, unsteady pressure drop across the face mask is measured for two different E:I = 1:1 (normal breathing) and 1:2 (inhalation time is twice the exhalation time). The breathing profiles for different E:I ratios are shown in Fig. 7(a), whereas the corresponding static pressure measured inside the nozzle fitted to the headform nose is shown in Fig. 7(b). The inhale duration is twice the exhale duration for E:I = 1:2, whereas for E:I = 1:1, the inhale and exhale duration is the same [Fig. 7(a)]. This distinct feature is also reflected in the static pressure variation shown in Fig. 7(b).

The unsteady static pressure is also measured for various popularly used single masks (two-layered cotton, surgical, five-layer, and N-95) and double masks (surgical + cotton, surgical + N-95, cotton + five-layer, and cotton + N-95). The mean and peak pressures for inhalation and exhalation are tabulated in Table I. The mean and peak pressures are normalized with the mean and peak pressures without mask case to generalize the results. Among the various widely used single masks tested in the present study, the N-95 mask showed the most promising results with just 17% and 14% higher mean and peak pressure drop compared to no mask case. A two-layer cotton mask incurs 61% and 46% higher mean and peak pressure drop; however, these values are significantly higher (780% and 870%) for a medical-grade surgical mask. The higher pressure drop across surgical and cotton masks over an N-95 mask is attributed to the construction and fitment of these masks. Cotton and surgical masks directly fit on the nose and block the airflow very close to the exit of the nose. Since the velocity at the exit of the nose is highest, hence these masks offer high-pressure drop. A standard N-95 mask has an extended profile, which does not block the air at the nose exit; hence, they offer low-pressure drops. The mean pressure drop for the surgical mask (105 Pa) and two-layer cotton mask (21.5 Pa) in the present case closely match with the pressure drop reported under steady flow conditions 196 and 30 Pa, respectively.^50,54^ The difference is mainly attributed to the fact that in the standard technique, the face mask material is mounted perpendicular to the airflow direction and is perfectly sealed to avoid leakage. In the present case, the face mask is snugly fitted onto the face of the headform, and due to the curvature of the face and nose, there is a natural gap between the face mask and the nose exit. Hence, air may leak sideways, and complete airflow may not be perpendicular to the face mask at the nose exit. This is responsible for lower pressure drop in the present case compared to the standard technique. However, the present technique resembles the actual scenario, and hence reported pressure drops are more realistic. The pressure drop reported for the N-95 mask (15 Pa) is very low compared to the pressure drop (210 Pa) reported using the standard technique,^50^ since, in the present technique, the extended profile of the N-95 mask does not block the air at the nose exit; hence, they offer low-pressure drop, whereas in the standard technique N-95 mask material directly blocks the air at the exit. In the present case, minor flow also leaks from the periphery of the mask due to the mask fitment on the face. In the actual scenario, the face mask also sits snugly on the face chin. However, it is practically not completely sealed; hence, the flow may leak from the mask periphery.

**TABLE I.** Mean and peak pressure for various masks for different E:I ratios.

	Inhalation				Exhalation			
	Mean pressure		Peak pressure		Mean pressure		Peak pressure	
E:I Mask	1:1	1:2	1:1	1:2	1:1	1:2	1:1	1:2
Cotton mask	−1.61	−1.46	−1.50	−1.46	1.73	1.65	1.73	1.63
Surgical mask	−7.88	−7.22	−8.79	−9.50	4.52	8.32	6.16	10.86
5 layer mask	2.71	2.47	2.47	2.22	2.9	2.94	2.87	2.57
N-95 mask	−1.17	−1.10	−1.14	−1.09	1.20	1.20	1.19	1.13
Surgical + cotton mask	−9.09	−8.26	−9.62	−10.04	7.05	12.94	11.02	16.59
Surgical + 5 layer mask	−11.43	−12.05	−11.66	−12.1	17.51	27.28	19.72	26.61
Surgical + N-95 mask	−8.30	−7.69	−9.10	−9.00	5.10	8.67	7.19	10.50
Cotton + 5 layer mask	−4.68	−3.8	−8.07	−8.57	3.62	5.62	10.27	13.73
Cotton + N-95 mask	−4.11	−3.55	−7.39	−5.29	1.88	3.01	4.45	6.01

**FIG. 8.**
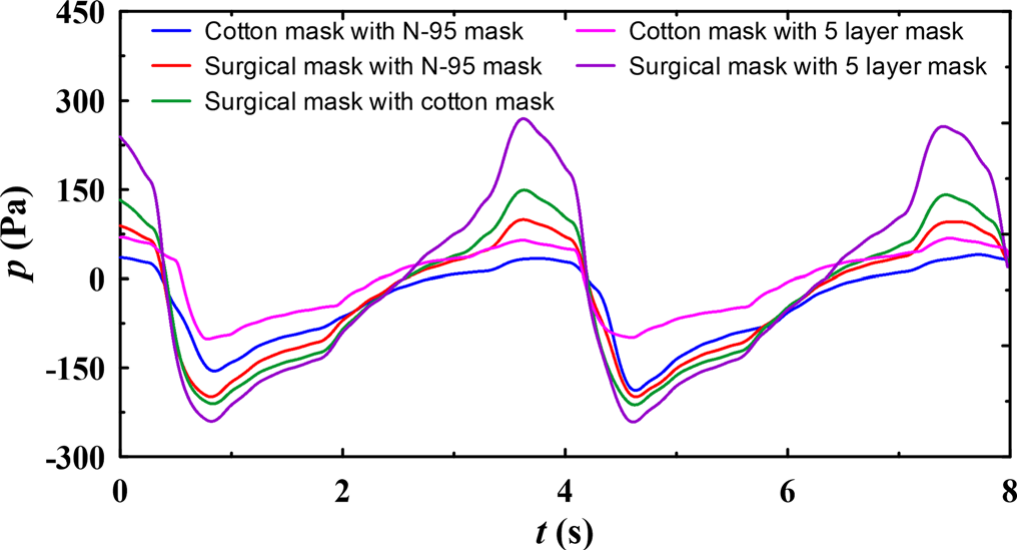
Static pressure variation for E:I = 1:1.

The pressure drop reported with double masking showed interesting results. A combination of surgical and N-95 masks offers 8.6% and 5.3% lower mean and peak pressures than surgical and cotton masks suggested by Brooks *et al.*^44^ However, the best results are observed with cotton, and N-95 masks with 54.6% and 23% lower mean and peak pressures than surgical and cotton masks. The double mask combination of surgical and cotton mask blocks the airflow at the exit of the nose; hence, they offer high-pressure drop. The pressure profiles for various mask combination for different E:I ratio are shown in Fig. 8. It is also interesting to observe that the mean and peak pressure are comparatively lower for E:I = 1:2 compared to E:I = 1:1 during inhalation, whereas during exhalation E:I = 1:1 offers lower pressure drop compared to E:I = 1:2, this is attributed to the nature of flow profiles as shown in Fig. 7. The peak flow rate for E:I = 1:2 is higher during exhalation and lower during inhalation as compared to E:I = 1:1. From the breathability perspective, which is related to health effects, cotton and N-95 double masking is the preferable choice. However, the filtration efficiency of this combination needs to be experimentally evaluated to establish this combination as the preferable double masking.

The present study highlights that a specific combination of double masks offers lower pressure drops than surgical masks. Hence, a proper choice of the double mask provides high protection and breathing comfort, which is essential to ensure both public health and curb the virus's spread. The present study can be extended further by simultaneous measurement of particle filtration efficiency and unsteady pressure drop across various double masks to establish double-masking efficacy. In addition, a detailed statistical correlation needs to be established between the pressure drop and physiologic and psychologic adverse health effects of long-term double mask usage.

## CONCLUSIONS

Double masking is an effective technique to improve mask fitment and protection. In the present work, flow visualization experiments demonstrated that a simple knot could improve the fitment of a surgical mask and minimize the leakage of the droplets. The leakage of the droplets is significantly reduced with a double mask, and a combination of surgical and N-95 masks completely impedes the leakage of the droplets in the forward direction. The breathability aspect of the face mask is quantified with unsteady static pressure measurements for different E:I ratios. A double mask combination of surgical and N-95 masks offers 8.6% and 5.6% lower mean and peak pressures than surgical and cotton masks. The best results are observed with cotton and N-95 masks with 54.6% and 23% lower mean and peak pressures than surgical and cotton masks. The filtration efficiency of this combination needs experimental evaluation to establish this combination as preferable double masking. These results help assess the physiologic and psychologic adverse health effects associated with long-term mask usage.

## Data Availability

The data that support the findings of this study are available from the corresponding author upon reasonable request.
